# Effects of Electrical Parameters on Micro-Arc Oxidation Coatings on Pure Titanium

**DOI:** 10.3390/mi14101950

**Published:** 2023-10-19

**Authors:** Aqeel Abbas, Hsuan-Ping Kung, Hsin-Chih Lin

**Affiliations:** Department of Materials Science and Engineering, National Taiwan University, Taipei 10617, Taiwan; engr.aqeel14@gmail.com (A.A.);

**Keywords:** pure titanium, micro-arc oxidation (MAO), microstructures, wear test, electrochemistry

## Abstract

The micro-arc oxidation process was used to apply a ceramic oxide coating on a pure titanium substrate using calcium acetate and sodium dihydrogen phosphate as an electrolyte. The influence of the current frequency and duty ratio on the surface morphology, phase composition, wear behavior, and corrosion resistance were analyzed by employing a scanning electron microscope, X-ray diffractometer, ball-on-disk apparatus, and potentiodynamic polarization, respectively. Analyses of the surface and cross-sectional morphologies revealed that the MAO films prepared via a low current frequency (100 Hz) and a high duty ratio (60%) had a lower porosity and were more compact. The medium (500 Hz) and high (1000 Hz) frequencies at the higher duty ratios presented with better wear resistance. The highest film thickness (11.25 µm) was achieved at 100 Hz and a 20% duty ratio. A negligible current density was observed when the frequency was fixed at 500 Hz and 1000 Hz and the duty cycle was 20%.

## 1. Introduction

Titanium and its alloys are widely used in the aerospace, marine, and biomedical industries because of their high strength-to-weight ratio, chemical stability, excellent corrosion resistance, and good performance at high temperatures [1,2]. Titanium is 60% lighter than steel, and its strength is equivalent to low-carbon steel [3]. The physical and chemical properties of pure titanium can be improved by adding different alloying elements; these alloying elements also change the phase transformation temperature of pure titanium. The standard electrode potential for Ti is −1.63 V, which means it has a great affinity for oxygen, and that a chemically stable oxide film will be formed on its surface [4,5,6]. In addition, if the oxide film on the surface of the titanium alloy ruptures in a corrosive environment, crevice corrosion may occur [7,8,9]. Many surface-modification techniques such as atomic layer deposition, plasma electric oxidation, anodic spark deposition, and micro-arc oxidation are carried out to overcome these shortcomings [10,11].

Micro-arc oxidation (MAO) is a cost-effective way of improving mechanical strength and corrosion resistance [12]. Micro-arc oxide formation is the electrochemical process of generating a ceramic oxide film on a metallic substrate [2]. MAO is the technique of applying a high voltage between the electrode and the examined metal. The MAO film is influenced by many parameters, such as the choice of substrate, the time of the micro-arc oxidation, the configuration of the electrolyte system, and the adjustment of power supply parameters [1]. The MAO film will grow linearly up to a certain time, and too long of a time will cause a deviation from this linear growth. The electrolyte composition and concentration affect the structural composition, growth rate, and density of the micro-arc oxidation film. Sarbishei et al. [13] investigated the effects of different electrolyte concentrations under constant electrical parameters and observed different surface morphologies. An increase in the current frequency leads to an increase in the number of alternations, which means that the positive current of each cycle acts on the anode for a short time [14]. An increase in the duty cycle of the anode pulse will increase the pulse energy and spark discharge intensity on the film surface, which further enhances the grain size and surface roughness. A higher duty cycle increases the discharge energy, which induces the anatase phase transformation to form rutile [15]. Rapheal et al. [16] and Wang et al. [17] found that a higher anodal current density increases the film thickness and surface roughness, but decreases the microhardness.

The power supply mode consists of a direct current, unipolar pulse, and bipolar pulse. The power mode of the bipolar pulse can adjust the pulse waveform to form an alternating current with different positive and negative bias voltages. The alternating discharge in the bipolar pulse mode allows us to determine the discharge formation and the discharge interruption time in the micro-arc discharge process [18].

MAO is one of the most common and economical ways of developing titanium dioxide films on Ti surfaces. The most common crystals of titanium dioxide are mainly divided into three types: Anatase, Rutile, and Brookite. Anatase and rutile belong to the tetragonal system, while brookite belongs to the orthorhombic system [18,19]. All three crystals are made of a composition of titanium oxide (TiO_2_) octahedrons, but their physical properties are different due to how the atoms are connected. Brookite is the least common in nature, while anatase and rutile are more widely used phases [20]. The excellent performance of MAO coatings makes them more suitable for applications in biomedical fields, automobiles, and the defense industry. However, they still have shortcomings that limit their applications; for example, a high voltage (400 to 700 V) and high current density (10 to 60 A/dm^2^) are commonly used to maintain the micro-arc state on the metal surface, and the cost of this is very high. Therefore, MAO technology is applied to small workpieces to control the energy consumption to a reasonable cost level.

C.G-Yu et al. [21] showed that TiO_2_ films are completely composed of rutile anatase phases, and that the film thickness is directly related to the duty cycle and voltage; the surface roughness and pore size were also increased, but the pore density was decreased. The corrosion resistance of the film was also closely linked with the voltage and duty cycle. Magda Dziaduszewska et al. [22] investigated TiO_2_ films via the MAO process under different electrical parameters and found that voltage had the greatest influence. Yu-Fang Hong et al. [23] formed a dark-color homogeneous TiO_2_ film on pure titanium via the MAO process under different electrical parameters; the surface was hydrophobic and had excellent corrosion resistance. Chai et al. [24] analyzed the effects of electrical parameters on MAO film formation on aluminum substrates. They found that the hardness and thickness of the film were affected in the manner of forward voltage > forward duty cycle > pulse frequency. The electrical parameters affected the corrosion resistance in the order of pulse frequency > forward voltage > forward duty cycle.

The majority of this research work has been performed to analyze the effects of the electrolyte composition on the structure and properties of MAO coatings. However, the correlation between electrical parameters and MAO film formation and its characteristics when formed on pure titanium has not been investigated.

Therefore, the objective of the present work is to investigate the effects of the bipolar current, frequency, and duty cycle of the MAO process on coating formation on pure titanium. The surface morphology, chemical composition, and corrosion behavior of the film have been examined in detail.

## 2. Experimental Methods and Procedure

### 2.1. Sample Preparation

Pure titanium (grade-4) was provided by Huangchieh Metal Composite Material Technology Co., Ltd. Specimens with dimensions of 30 mm × 25 mm × 2 mm were ground using 120, 400, 800, 1200, and 2500 grit-sized silicon carbide sandpapers. The samples were polished with a 0.3 μm alumina suspension to obtain mirror-like surfaces. A screw thread (M5) was drilled into the specimen to connect the electrode hanger. The gaskets were placed at the joints between the specimen and the electrode to prevent the leakage of electricity during the MAO process. The specimens were cleaned in an ultrasonic oscillator with deionized water and alcohol prior to the micro-arc oxidation process to prevent the surface from being stained.

### 2.2. Micro-Arc Oxidation Film Formation

The experimental setup for the micro-arc oxidation consisted of an electrolysis cell, air pumping system, cooling circulation system, power supply, pulse rectifier, computer control system, and air extraction equipment. An electrolyzer tank with a 2-L volume was used as a cathode. The MAO process was carried out using an SY-P10600200 power supply with the following electrical parameters—I_max_: 10 A and U_max_: 600 V. The power supply provided the waveform of the bipolar pulse mode. The cooling circulation system was used to isolate the electrolyte for cooling, and an air blower was used to blow bubbles into the electrolyte to circulate the electrolyte evenly. The uniform circulation of the electrolyte can make the electrolyte–specimen contact even and quickly dissipate the heat generated in the micro-arc oxidation (MAO) reaction.

The electrolyte for all the parameters in this experiment was made of an equal amount of calcium acetate Ca(C_2_H_3_O_2_)_2_·H_2_O and sodium dihydrogen phosphate (NaH_2_PO_4_). The MAO treatment time for all parameters was 10 min. The anodic and cathodic current densities were fixed at 15 A/dm^2^ and 2 A/dm^2^, respectively. The parameters and codes for different duty cycles and frequencies are shown in Table 1. The anode and cathode discharge time and off time for each parameter are given in Table 2.

### 2.3. Microstructural Observation and Surface Roughness

A scanning electron microscope with an EDS detector (JEOL JSM6510, Tokyo, Japan, accelerating voltage 0.5–30 kV, magnification 5×–300,000×) was used to analyze the surface and cross-section of the MAO film. An X-ray diffractometer (XRD; Rigaku TTRAX 3, Rigaku Co., Ltd., Tokyo, Japan) using a characteristic wavelength of λ = 1.542 Å, a scanning rate of 1.4 degrees/min, and a diffraction angle 2θ range of 20° to 80° was employed to analyze the phase composition. The operating voltage of the diffractometer was set at 15 kV, and the operating current was 300 mA. The average center line roughness (Ra) was measured using the machine (model Mitutoyo SJ-201, Mitutoyo Co., Aurora, IL, USA).

### 2.4. Pin-on-Disc Wear Test

The wear resistance of the MAO films was measured by a wear test machine (mode: CSM TRB 01-05600 Nano-Tribometer, made by CSM Instruments, Peuseux, Switzerland). According to the ASTM G99-17 test standard [25], the wear test was performed against a tungsten carbide ball (hardness: 90HRA) in a pin-on-disk mode, under a load of 3 N at a fixed rotation speed of 0.1 m/s and 6000 cycles with a 12 mm rotation diameter. Then, the friction coefficient and wear rate were measured. The wear track was observed using a scanning electron microscope (JEOL JSM6510, JEOL Ltd, Tokyo, Japan), and the wear track depths were measured by a laser scanning microscope (VK-9710, KEYENCE America, Elmwood Park, NJ, USA).

### 2.5. Corrosion Resistance Analysis

A potentiostat (Multi Autolab/M204, made by Metrohm Company, Herisau, Switzerland) was utilized to investigate the corrosion resistance of the MAO film in a 3.5 wt% NaCl solution (electrolyte). The system employed a saturated calomel electrode (SCE) as the reference electrode, a platinum sheet as the counter electrode, and the sample as the working electrode. To assess the electrochemical properties of the MAO coating, potentiodynamic polarization curves (Tafel curves) were generated. Prior to the measurement of the polarization curves, the Open Circuit Potential (OCP) was set for an hour. The Tafel curves, which represent the potentiodynamic current-potential curves, were recorded using a scan rate of 1 mV/s, ranging from 3 V to 0.5 V.

## 3. Results and Discussion

### 3.1. Process Characterization

**Figure 1:**
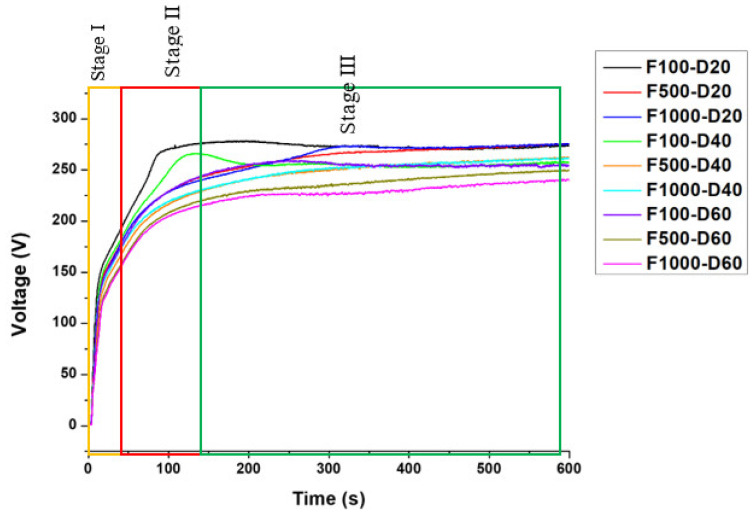
Voltage–time curve during the process of micro-arc oxidation.

The voltage-time plots of the analyzed samples at various frequencies and duty ratios are displayed in Figure 1. Three primary stages are evident from the figure. During stage I, the voltage quickly increased to 125 V. At this stage, an amorphous layer forms on the surface and gas bubbles emerge, as reported by Dehnavi et al. [26]. The amorphous layer is created on the surface by the release of titanium ions into the electrolyte when the current is t_on_. The voltage increases gradually during stage II, and dielectric breakdown occurs with uniformly appearing electrical sparks on the surface. At this stage, the transition from an amorphous to a crystalline structure of the oxide coating takes place [14]. Oxygen ions interact with Ti ions to form an amorphous TiO_2_ film that transforms into a crystalline phase. The newly formed TiO_2_ coating undergoes compaction during subsequent dielectric breakdown [27]. The voltage stabilizes almost entirely during stage III. As the spark intensifies, the charge transfer resistance increases. The voltage during MAO formation is automatically adjusted by the power supply, with fixed cathode and anode current densities. The voltage–time plots of the MAO process, displayed in Figure 1, are significantly influenced by the variation of the duty ratio and frequency [15]. Increasing the duty ratio from 20% to 60% decreased the voltage from 275 V to 220 V after 10 min of the MAO process. During stage II, the film formation process was found to be faster for F100-D20 and F100-D40, as evidenced by a steeper slope in their respective voltage–time plots in comparison to the other samples. When the frequency was fixed at 100 Hz, the final voltage difference between the duty ratios of 20% and 60% was the smallest at 20 volts, while the largest final voltage gap of 35 volts occurred when the frequency was fixed at 1000 Hz. During stage I, an amorphous layer was formed on the surface via the release of titanium ions into the electrolyte when the current was t_on_. Micro-arc discharge occurs above the dielectric breakdown potential when the current is supplied with an anodic polarization [28]. The MAO process is accompanied by a strong electric field, high temperature, and pressure. The strong electric field affects the diffusion of oxygen ions towards the Ti substrate [29].

**Figure 2:**
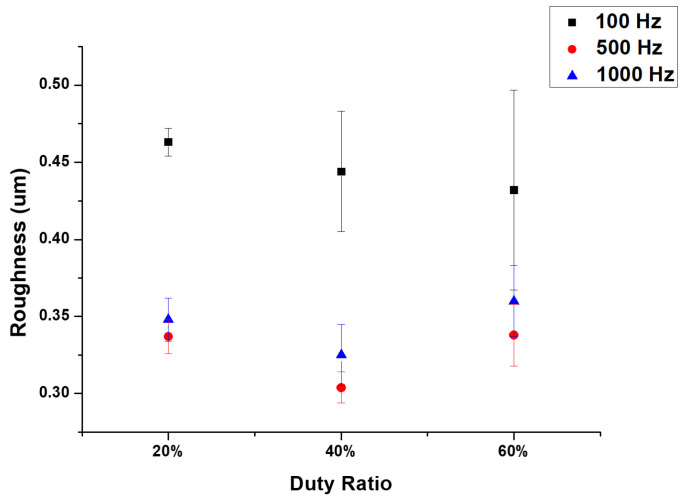
Surface roughness of MAO coatings at different frequencies and duty cycles.

The surface roughness for each parameter is displayed in Figure 2. It is evident from the figure that the roughness reached its maximum at a frequency of 100 Hz and was unaffected by the duty ratio. The surface roughness was slightly impacted by the duty cycle but was significantly influenced by the frequency. The F100-D20 samples showed the maximum surface roughness of 0.465 µm.

### 3.2. Surface Characterization

**Figure 3:**
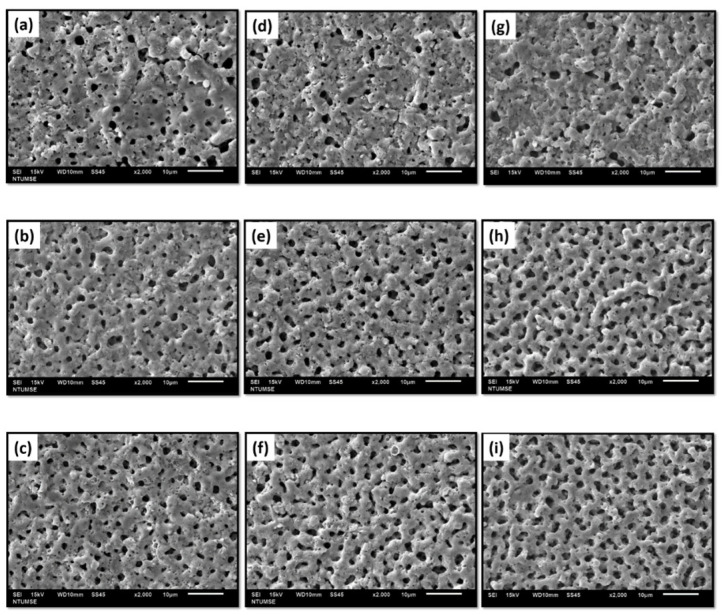
Microstructures of MAO coatings on different samples: (**a**) F100-D20; (**b**) F500-D20; (**c**) F1000-D20; (**d**) F100-D40; (**e**) F500-D40; (**f**) F1000-D40; (**g**) F100-D60; (**h**) F500-D60; (**i**) F1000-D60.

The microstructure of the MAO film at different parameters under a fixed current density is shown in Figure 3. The surface analysis uncovered numerous sub-micron pores in all the samples examined. These pores formed as a result of dielectric breakdown at extremely high temperatures. The high currents and voltages associated with MAO significantly affect the surface morphology [30]. The distribution of current and voltage over the surface is non-uniform and impacted by the coating’s dielectric properties. The formation of pores occurs spontaneously during dielectric breakdown and is dependent on the pore’s size and shape. Porosity can be controlled by varying the current and frequencies [31].

The surfaces processed at a low frequency had the smallest number of pores, regardless of duty ratio. This can be attributed to a longer t_on_^+^ in the overall discharge cycle. The high temperature and pressure caused by the extended micro-arc discharge time benefit the substrate, as the micro-arc flows up the small holes, resulting in a more sintered surface. Increasing the current frequency results in a larger number of alternating discharge cycles and a shorter time for the oxide film to act as an anode. Conversely, decreasing the current frequency increases the micro-discharge intensity, leading to a rise in the number of micro pores in the film [32].

The surface pores will shrink regardless of the duty ratio. At a frequency of 100 Hz and duty ratio of 60%, the number of surface pores decreased, attributed to the longer t_on_ at this frequency. This accumulation of heat or energy caused the deposited film to melt and fill the pores more easily. Adjusting the frequency to 500 Hz and 1000 Hz resulted in less energy being accumulated on the surface due to the shorter discharge period. This led to a smaller amount of deposited film melting and filling into the pores, resulting in higher porosity at higher frequencies.

**Figure 4:**
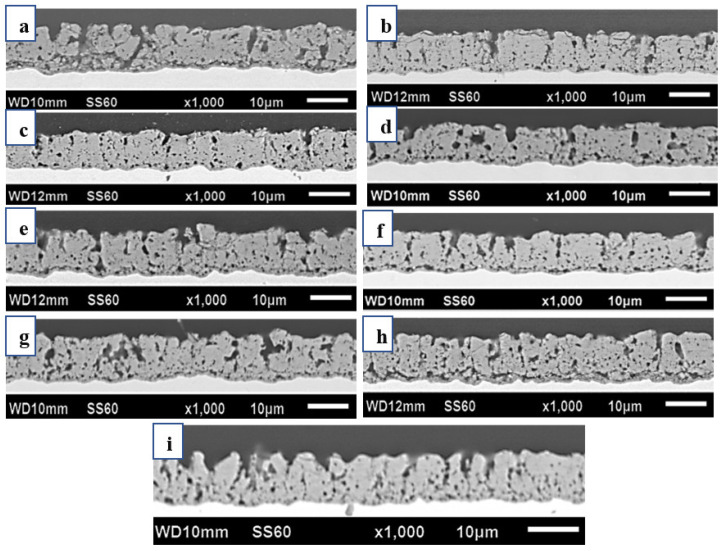
Cross-sectional images of MAO coatings on different samples: (**a**) F100-D20; (**b**) F500-D20; (**c**) F1000-D20; (**d**) F100-D40; (**e**) F500-D40; (**f**) F1000-D40; (**g**) F100-D60; (**h**) F500-D60; (**i**) F1000-D60.

Figure 3 shows a buildup of ablatives on the surfaces of the F100-D20, F500-D20, and F1000-D20, caused by the large discharge phenomenon. The surface morphology was smoother at a 40% and 60% duty ratio, but micro-cracks were present when processed at a 20% duty ratio. The MAO coating process generates spark discharges, leading to melting, sintering, and localized high temperatures based on the pulse supply. Figure 4 depicts the cross-sectional morphology of MAO coatings that were processed using different duty ratios and frequencies. Samples processed at a higher duty ratio and lower frequency exhibited pores and a loose coating.

**Figure 5:**
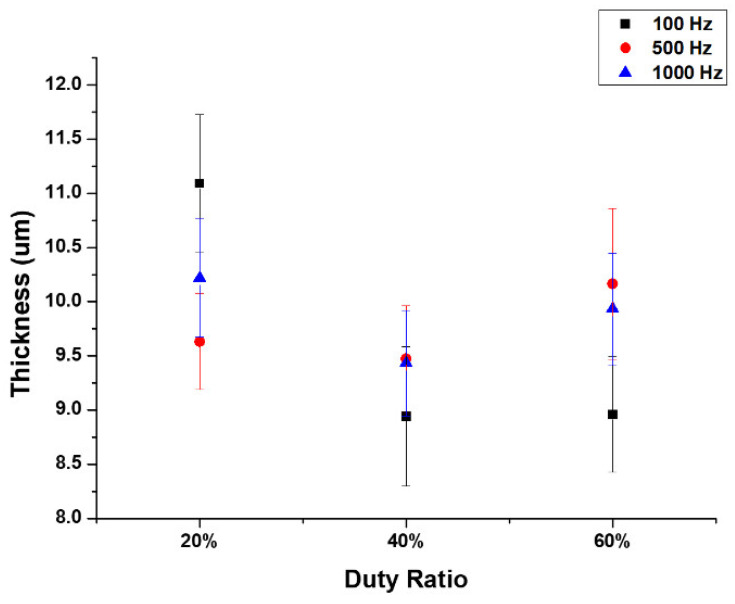
Film thickness of MAO coatings with changing frequencies and duty cycles.

The cross-sectional image of the film layer displays significant discharge channels that are predominantly large in size. At a fixed frequency of 100 Hz and a duty ratio of 20%, the connection between the dense inner and outer layers of the oxide film was reasonably substantial. At a duty ratio of 40%, numerous holes remained visible in the outer layer of the oxide film. Nevertheless, the film layer at the point of intersection with the dense inner layer appeared to be more continuous. The pores in the outer layer of the oxide film were significantly reduced when increasing the duty ratio to 60%. Furthermore, fewer holes were present at the junction of the dense inner layer and the outer layer of the oxide film. Figure 5 displays the MAO film thickness at various duty ratios and frequencies. The maximum film thickness (11.3 µm) was observed in the F100-D20 samples, reduced to 9 µm in F100-D60. The thickness of the film increased from 9.5 µm to 10.2 µm with an increase in duty ratio to 60%, at a constant frequency of 500 Hz.

It can be summarized that low-frequency and high-duty cycle micro-arc film structures are relatively less dense. When operating under the conditions of a low frequency and large duty cycle, the duration of the t_on_+ time will be the longest. Additionally, the long discharge arc time benefits the molten substrate ejection to the surface and the sintering of the oxide film layer, which is crucial to forming a film structure with fewer holes under low-frequency and high-duty cycle conditions [28].

**Figure 6:**
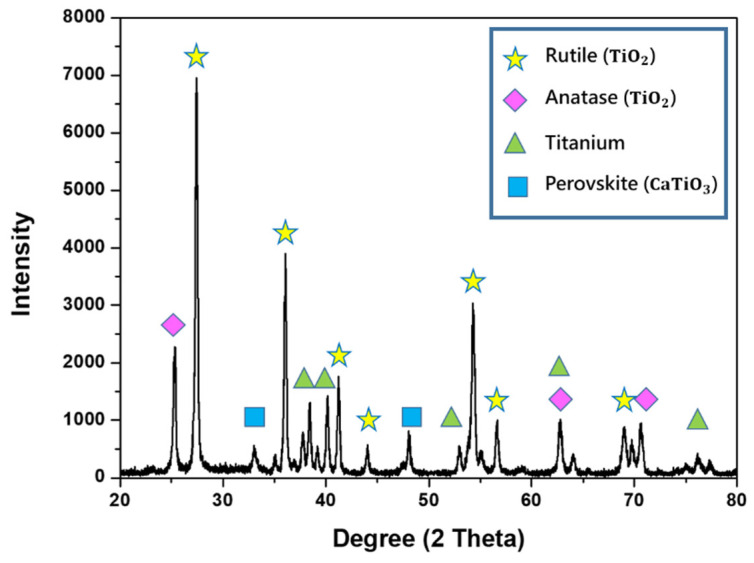
XRD diffraction pattern of F1000-D20.

The typical XRD pattern of a MAO coating on F1000-D20 is illustrated in Figure 6. The MAO coating was found to consist of rutile, anatase, and perovskite phases. Peaks related to Ti were also observed in XRD spectra. The phases of the rutile, anatase, and perovskite were formed by the spraying of molten oxide onto the surface during the MAO coating process. The XRD results show that using a bipolar current, varying the reaction temperature, and repeated remelting increases the complexity of the oxidation product. The intensity of the rutile peaks was much higher than those of the anatase and perovskite; this is because of the transformation from anatase to rutile that occurred when the process reached the dielectric breakdown temperature. The metastable phases are irreversible and transform into the rutile phase at a low frequency. X-ray analysis did not detect any additional phases. However, there may be some other substances in the coating that existed in a crystalline or amorphous structure, with a content level that fell below the limit of XRD detection.

### 3.3. Wear Resistance Performance

**Figure 7:**
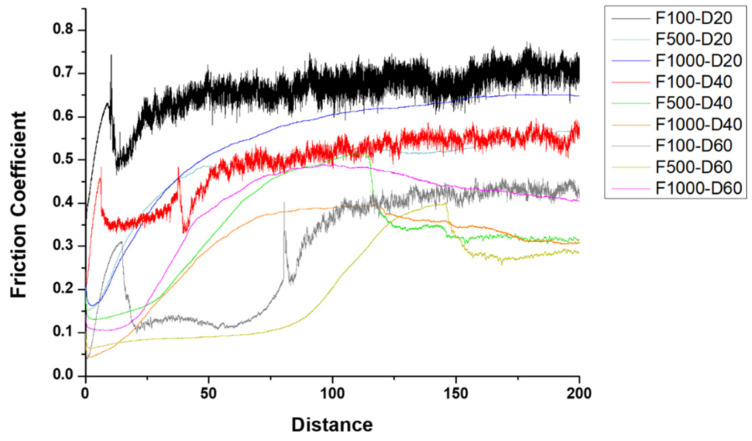
Friction coefficient curves for MAO samples under varying frequencies and duty cycles.

The friction coefficient curves of all the samples subjected to the ball-on-disk wear test are presented in Figure 7. The figure indicates a correlation between the friction coefficient and surface roughness, as illustrated in Figure 2. An increase in surface roughness results in a higher coefficient of friction. The trend in the friction coefficient can serve as a gauge for determining the time of the micro-arc oxidation film rupture. The rupture distance of the F100-D20, F100-D40, and F100-D60 was recorded as 13 m, 40.4 m, and 83.7 m, correspondingly. The remaining MAO samples subjected to a 200-m wear test did not exhibit any substrate exposure.

**Figure 8:**
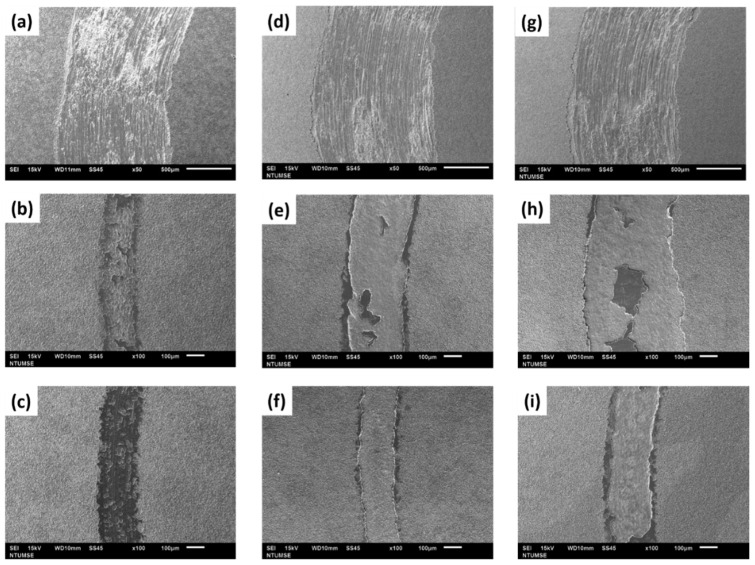
Surface microstructures of the MAO films examined after conducting a wear test under varying frequency and duty cycle conditions. (**a**) F100-D20; (**b**) F500-D20; (**c**) F1000-D20; (**d**) F100-D40; (**e**) F500-D40; (**f**) F1000-D40; (**g**) F100-D60; (**h**) F500-D60; (**i**) F1000-D60.

The surface morphologies of all the samples after the wear test, at varying frequencies and duty cycles, are exhibited in Figure 8. The micro-arc oxidation coatings of (a), (d) and (g) were the only ones completely worn away, as demonstrated in the figure. At a later stage of the wear test, only the tungsten steel grinding ball and the pure titanium base material exhibited wear behavior, resulting in severe scratching on the surface. The microstructure of the worn surface depicted in Figure 8b,e,f,h,i shows that a portion of the micro-arc oxidation film was removed, but it was not worn down to the pure titanium substrate. This suggests that the micro-arc oxidation film continued to fulfill its role in protecting the pure titanium substrate. Notably, the wear track width in Figure 8c is minimal, indicating that the wear test resulted in only slight abrasion damage.

The film’s wear rate was determined using wear depth data, the average track width, the sliding distance, and the abrasion load. Table 3 presents values for the crack width, depression depth, and wear rate.

After reorganizing the experimental data, the discussion can be divided into two parts. Firstly, an examination of the MAO film’s wear resistance capability by varying the frequency when the duty cycle is constant. Regardless of the duty cycle parameter, when the total wear distance was less than 100 m, the 100 Hz specimen exposed the substrate. However, the films fabricated in the high-frequency state of 500 Hz and 1000 Hz demonstrated excellent wear resistance. Even after a wear distance of 200 m, it could still protect the pure titanium substrate from wear.

The second part highlights how the MAO film can withstand wear by adjusting the duty cycle while keeping the frequency constant. At 100 Hz, the duty cycle can be altered between 20% and 60% to postpone the crack formation of the micro-arc oxide film. When the frequency was set at 500 Hz and 1000 Hz, the micro-arc oxide film wore away and all samples exhibited good wear resistance. However, by adjusting the duty cycle from 20% to 60%, the depression depth of the micro-arc oxide film increased. According to Table 3, when the frequency was 500 Hz and 1000 Hz, as the duty cycle decreased, the wear rate also decreased. Additionally, the micro-arc oxide film exhibited the lowest wear rate at a duty cycle of 20%, indicating the best wear resistance.

### 3.4. Corrosion Resistance

**Figure 9:**
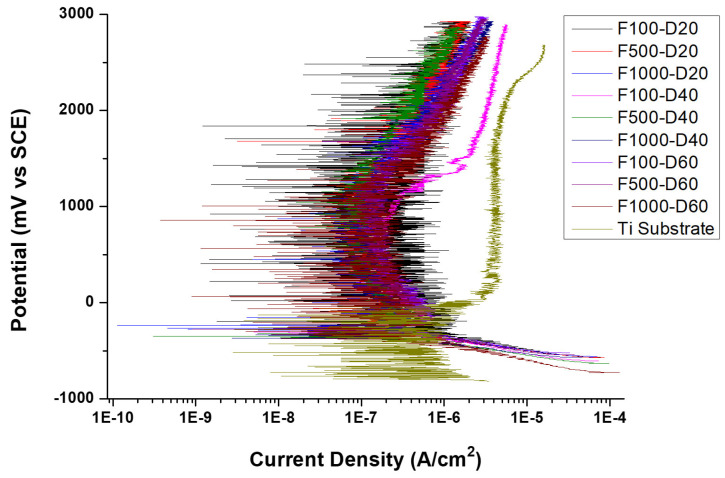
Polarization curve of micro-arc oxide films with varying frequencies and duty cycles.

The polarization curves of the micro-arc oxide films, with varying frequencies and duty cycles, are illustrated in Figure 9. It is evident that the polarization curves of the MAO film for distinct parameters and those of the pure titanium substrate exhibited similar trends. Table 4 presents the corrosion potential and corrosion current density of the films under different frequency and duty cycle parameters. The corrosion potential and current density for each parameter exhibited minimal differences, as illustrated by Figure 9 and Table 4. This can be attributed to the similarities in the properties of the passivation films developed on the pure titanium substrate.

Furthermore, the corrosion potential of the micro-arc oxidation film tended to increase when compared to the pure titanium substrate. The corrosion current density of a titanium dioxide film diminishes after micro-arc oxidation as compared to a pure titanium substrate [33]. Hence, it could be inferred that a micro-arc oxidation film covering a pure titanium substrate can improve its corrosion resistance. The film’s microporous nature may contribute to substrate corrosion [34].

Microporous corrosion can be broken down into three distinct stages: the nucleation of metastable micropores, the growth of these micropores, and the eventual passivation thereof or the transition to a steady-state pitting development. The polarization curves of the film that had undergone micro-arc oxidation exhibited current density oscillations (Figure 9). Such oscillations can be attributed to the ongoing formation and passivation of metastable pits [34].

Assuming the passivation film is in a non-corrosive ion environment, the Point Defect Model (PDM) indicates that the formation and breakdown of the passivation film is in a stable state of equilibrium. Nevertheless, an active anion environment (such as chloride ions) would disrupt the passivation film’s equilibrium state—primarily as chloride ions would selectively distribute themselves within the faulty passivation film, competing with oxygen ions. Finally, it reacts with the anions present in the passivation film, resulting in the formation of soluble chlorides. As a result, the dissolved state will be dominant and lead to the formation of metastable micropores [35]. However, during the late stage of metastable micropore growth, the passivation film may rupture due to osmotic pressure or stress; this leads to accelerated diffusion of the solution both within and outside the micropores. At this point, the corrosion solution within the micropores is no longer able to maintain its original high concentration state [36].

The corrosion current density depicted in Figure 9 has two parts for discussion. At first, when the duty cycle remained constant, an increase in frequency was likely to decrease the corrosion current density. Therefore, utilizing medium and high-frequency parameters for a fixed duty cycle was advantageous for enhancing the corrosion resistance of the film. This result is consistent with the cross-sectional images of the micro-arc oxide film. Fewer holes in the cross-sectional structure of the film suggest a higher level of resistance to corrosion [37].

When the frequency was fixed at 100 Hz and the duty cycle was set to 60%, the micro-arc oxidation film exhibited the smallest corrosion current density. Similarly, when the frequency was fixed at 500 Hz and 1000 Hz while the duty cycle was set at 20%, the micro-arc oxidation film showed the lowest corrosion current density. Thus, by using a medium to high frequency with a 20% duty cycle, the micro-arc oxide film demonstrates the best corrosion resistance [38].

## 4. Conclusions

1. The micro-arc oxidation film surface exhibited a porous microstructure. By fixing the frequency at 500 Hz and 1000 Hz, and adjusting the duty cycle from 20% to 60%, the surface porosity became more pronounced.
2. At a fixed frequency of 100 Hz and a duty ratio of 60%, the micro-arc oxide film’s structure was denser. The protracted discharge arc promoted the molten substrate’s eruption to the surface while also sintering the oxide film layer.
3. Implementing medium and high-frequency parameters enhanced the film’s wear resistance, as demonstrated by the cross-sectional microstructure of the micro-arc oxidation film. A lower porosity in the film resulted in higher resistance to abrasion.
4. The polarization curve of the micro-arc oxide film under all conditions showed a consistent pattern. By fixing the duty cycle, the utilization of medium and high-frequency parameters enhanced the resistance of the film against corrosion. Corrosion resistance was improved with fewer holes in the cross-sectional structure of the film. When set at a fixed frequency of 500 and 1000 Hz with a duty cycle of 20%, the micro-arc oxide film exhibited the lowest corrosion current density.

## Figures and Tables

**Table 1:** Parameters and codes of different frequencies and duty cycles.

	Duty Ratio	20%	40%	60%
Frequency				
100 Hz		F100-D20	F100-D40	F100-D60
500 Hz		F500-D20	F500-D40	F500-D60
1000 Hz		F1000-D20	F1000-D40	F1000-D60

**Table 2:** ${t_{on}}^{+}$, ${t_{off}}^{+}$, ${t_{on}}^{-}$ and ${t_{off}}^{-}$ time of each parameter.

	Time	${t_{on}}^{+}$ (μs)	${t_{off}}^{+}$ (μs)	${t_{on}}^{-}$ (μs)	${t_{off}}^{-}$
Sample					
F100-D20		2000	2500	3000	2500
F100-D40		4000	2500	1000	2500
F100-D60		6000	1500	1000	1500
F500-D20		400	500	600	500
F500-D40		800	500	200	500
F500-D60		1200	300	200	300
F1000-D20		200	250	300	250
F1000-D40		400	250	100	250
F1000-D60		600	150	100	150

**Table 3:** Wear width, depth, and rate of MAO films under different frequencies and duty cycles.

Frequency	20%			40%			60%		
	Width (μm)	Depth (μm)	Rate mm^3^/(m.N)	Width (μm)	Depth (μm)	Rate mm^3^/(m.N)	Width (μm)	Depth (μm)	Rate mm^3^/(m∙N)
500 Hz	214.028	2.646	1.179 × 10^−5^	366.415	8.813	1.014 × 10^−4^	447.608	9.146	1.286 × 10^−4^
1000 Hz	207.310	0.805	5.243 × 10^−6^	250.911	1.783	1.405 × 10^−5^	251.906	4.304	3.406 × 10^−5^

**Table 4:** Corrosion potentials and corrosion current densities of MAO films with varying frequencies and duty cycles.

Sample	Corrosion Potential (mV)	Current Density (A/cm^2^)
F100-D20	−264.740	1.320 × 10^−5^
F500-D20	−266.876	4.588 × 10^−6^
F1000-D20	−266.571	2.618 × 10^−6^
F100-D40	−306.702	7.272 × 10^−6^
F500-D40	−348.053	6.114 × 10^−6^
F1000-D40	−367.126	4.795 × 10^−6^
F100-D60	−276.337	6.304 × 10^−6^
F500-D60	−274.658	6.314 × 10^−6^
F1000-D60	−272.980	6.195 × 10^−6^
Ti Substrate	−514.984	9.500 × 10^−5^

## Data Availability

Data will be made available based on a request to the authors.
